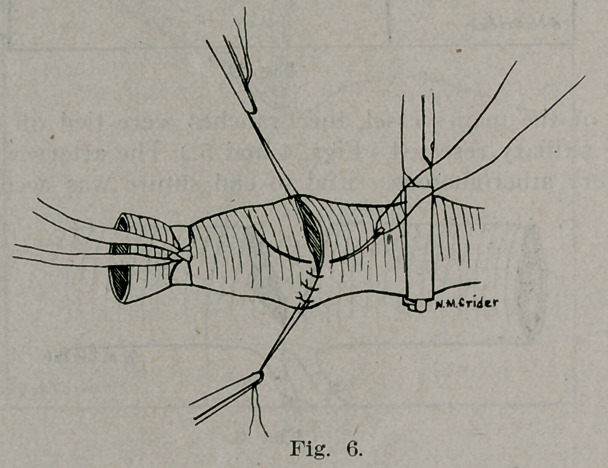# Circular Resection and Suture of the Axillary Artery for Transverse Laceration by Fracture-Dislocation of Anatomical Neck of the Humerus

**Published:** 1913-03

**Authors:** 


Circular Resection and Suture of the Axillary Artery for Transverse Laceration by Fracture-Dislocation of Anatomical Neck of the Humerus. J. J. Buchanan, of Pittsburgh, *Surgery, Gynecology,* and *Obstetrics,* December, 1912. The case reported, of almost complete transverse laceration of the axillary artery, is unique in its causation. The patient is a man of 58, who sustained a fracture-dislocation of the anatomical neck of the humerus, by falling from a train. The diagnosis of the bony lesion was made with the X-ray, and an incision opening the axilla was made for removal of the entirely separated head. (Fig. 1.)

Fig. 1.

Gentle lifting of the head from its impacted position on the axillary vessels and nerves was followed by a spurt of blood, which revealed the laceration of the axillary artery. (Fig. 2.)

The laceration was immediately opposite the origin of the subscapular and posterior circumflex branches. After temporary

Fig. 2.

control of the main vessel, the branches were tied off (Fig. 3) and the axillary resected (Figs. 4 and 5.) The artery was found to be very atheromatous. End to end suture was accomplished

Fig. 3.

by the method of Carrel (Fig. 6) with the exception that only two guy sutures were used and interrupted stitches applied.

The patient made a satisfactory recovery, although some spasticity of the arm remains and some limitation of the shoulder movements.

The writer has collated the literature of circular arterio-arterial suture in man and finds but twenty-nine other cases recorded.

By an analysis of these thirty cases he arrives at the following conclusions:

Fig. 4.

Fig. 5.

Fig. 6.

The small number of cases of circular arterial suture reported to this date can be accounted for in a variety of ways:

1. The fear of consecutive or secondary haemorrhage;

2. The expectation of weakening of the vessel with resultant aneurism;

3. The idea that the operation is difficult and requires special skill or previous animal experimentation and special instruments;

4. The belief that thrombosis is to be expected; which will put the procedure on a par with ligation;

5. The opinion that atheroma contra-indicates arterial suture. This has been emphasized by numerous surgeons, notably Forgue of Montpellier, Monod and Vanverts and von Schmieden of Berlin;

6. The fact that ligation, in a very large proportion of cases is followed by development of the collateral circulation and a result equal to arterial suture.

Taking up these points in detail, the following facts should be considered:

1. Haemorrhage has never followed circular arterial suture, and but one of the reported cases of lateral suture. This is probably due to the reason that, where perfect primary union is not secured by the accurate suture, thrombosis will promptly occur, which will sufficiently plug the vessel before the stitches could possibly give way.

2. Aneurismal dilatation has never been observed after arterial suture; this has been shown by an extensive inquiry by Matas.

3. The operation on vessels of the size of the femoral and axillary is no more difficult than many others that the general surgeon daily performs. Animal experimentation to acquire dexterity is certainly desirable but not essential to success for the ordinary operator. Instruments required are only such as are present in every well-equipped operating room.

4. Thrombosis undoubtedly occurs after many arterial sutures; but in the interim, the sutured vessel remains patulous and performs its function till its gradual closure by a thrombus has induced dilatation of the collateral vessels.

5. The writer's case is but one among a number which shows that no harm may result from arteriorrhaphy in vessels markedly atheromatous.

6. Gangrene occurs in a certain proportion of cases of ligature of main arterial trunks (stated by Wolf as fifteen per cent in the cases of axillary ligation) and certainly some of these unfortunate results can be saved by circular suture.

## Figures and Tables

**Fig. 1. f1:**
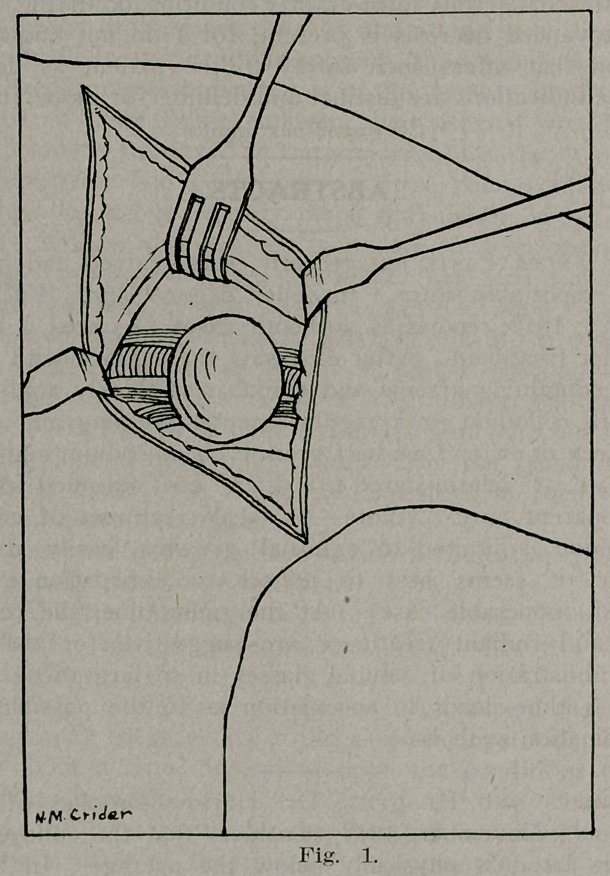


**Fig. 2. f2:**
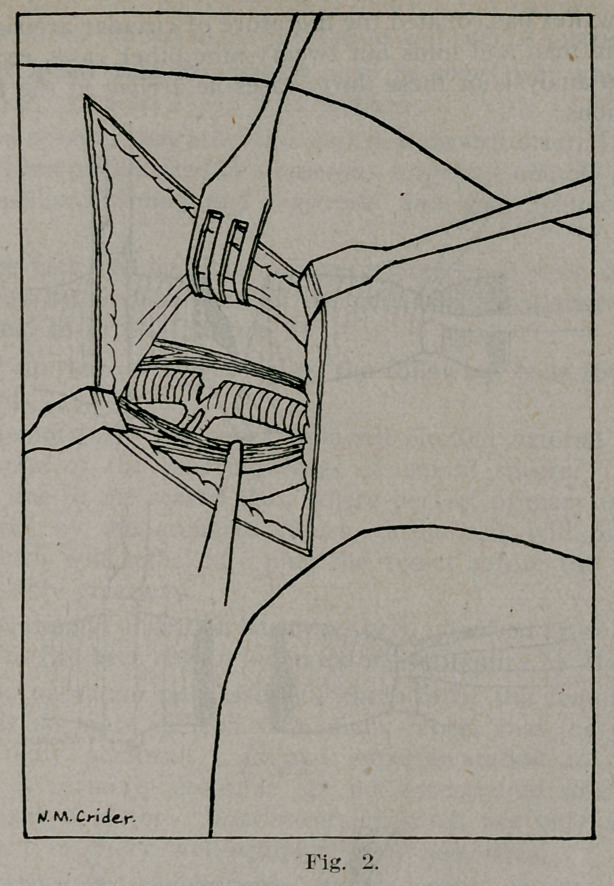


**Fig. 3. f3:**
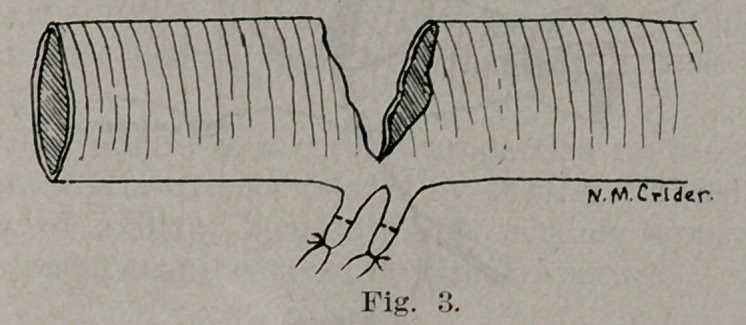


**Fig. 4. f4:**
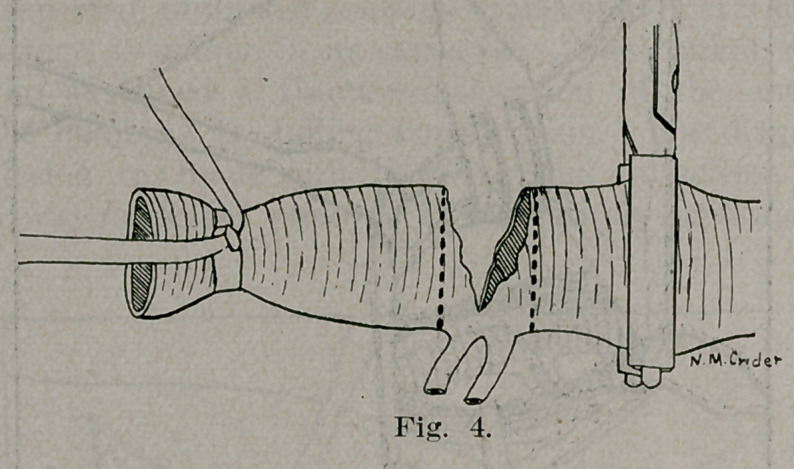


**Fig. 5. f5:**
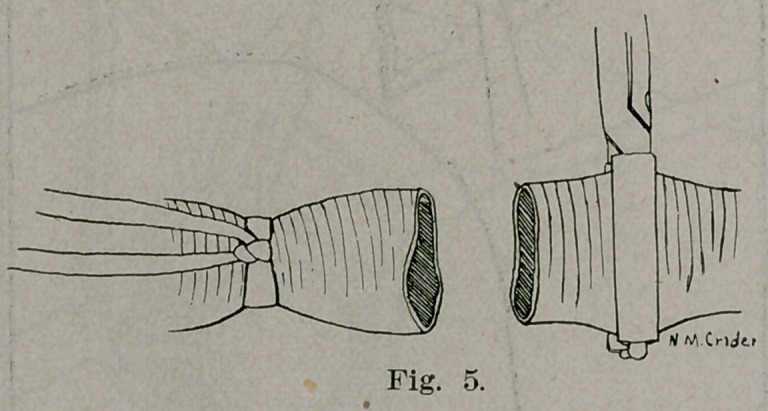


**Fig. 6. f6:**